# Exploring the conformational dynamics and key amino acids in the CD26-caveolin-1 interaction and potential therapeutic interventions

**DOI:** 10.1097/MD.0000000000038367

**Published:** 2024-05-31

**Authors:** Xiaopeng Hu, Chunmei Jiang, Yanli Gu, Xingkui Xue

**Affiliations:** a Medical Research Center, People's Hospital of Longhua, Shenzhen, China.

**Keywords:** alanine scanning, CD26-Caveolin-1 interaction, key amino acids, molecular dynamics simulations, potential therapeutic interventions

## Abstract

This study aimed to decipher the interaction between CD26 and caveolin-1, key proteins involved in cell signaling and linked to various diseases. Using computational methods, we predicted their binding conformations and assessed stability through 100 ns molecular dynamics (MD) simulations. We identified two distinct binding conformations (con1 and con4), with con1 exhibiting superior stability. In con1, specific amino acids in CD26, namely GLU237, TYR241, TYR248, and ARG147, were observed to engage in interactions with the F-J chain of Caveolin-1, establishing hydrogen bonds and cation or π–π interactions. Meanwhile, in con4, CD26 amino acids ARG253, LYS250, and TYR248 interacted with the J chain of Caveolin-1 via hydrogen bonds, cation–π interactions, and π–π interactions. Virtual screening also revealed potential small-molecule modulators, including Crocin, Poliumoside, and Canagliflozin, that could impact this interaction. Additionally, predictive analyses were conducted on the potential bioactivity, drug-likeness, and ADMET properties of these three compounds. These findings offer valuable insights into the binding mechanism, paving the way for new therapeutic strategies. However, further validation is required before clinical application. In summary, we provide a detailed understanding of the CD26 and caveolin-1 interaction, identifying key amino acids and potential modulators, essential for developing targeted therapies.

## 1. Introduction

Cluster of Differentiation 26 (a protein marker) (CD26), also known as dipeptidyl peptidase-4 (DPP4), is a 110-kDa transmembrane glycoprotein belonging to the prolyl oligopeptidase family. It is widely expressed in various cell types and tissues and has diverse biological functions, such as regulation of glucose homeostasis, modulation of immune responses, and activation of signaling pathways triggered by hormones and peptides.^[1]^ CD26 contains an extracellular region, transmembrane domain, and cytoplasmic tail that interacts with multiple proteins and regulates its enzymatic activity and signaling functions. CD26 exists in two forms: a membrane-bound receptor and a soluble form.^[2]^ Both forms of CD26 share the same catalytic domain and are involved in a variety of physiological processes such as glucose homeostasis, immune response regulation, and cell adhesion. However, there are several differences between these two forms. The membrane-bound form of CD26 is anchored to the plasma membrane through a glycosyl-phosphatidylinositol (GPI) linkage, which allows its interaction with other membrane-associated proteins such as Caveolin-1.^[3]^ In contrast, the soluble form lacks a GPI anchor and is released into the extracellular environment through various mechanisms, such as proteolytic cleavage and alternative splicing. Another difference between the two forms is their tissue distribution. The membrane-bound form is primarily expressed on the surface of various cell types, such as T cells, endothelial cells, and epithelial cells, whereas the soluble form is found in plasma and other bodily fluids.^[4]^ In addition, these two forms may have distinct roles in certain diseases. For example, the soluble form of CD26 has been shown to have pro-inflammatory effects, whereas the membrane-bound form may play a role in cell adhesion and migration. Overall, both forms of CD26 play important roles in various physiological processes; however, their distinct characteristics suggest that they may have unique functions and potential therapeutic implications.

CD26 has been shown to interacts with a variety of ligands, including incretins, chemokines, neuropeptides, and extracellular matrix proteins, and modulates their bioactivity and half-life. In addition, CD26 is involved in T cell activation, migration, and adhesion, and has been implicated in the pathogenesis of autoimmune diseases and cancer. Caveolin-1 is a scaffolding protein that is abundant in caveolae and is a specialized membrane domain that plays a role in cellular signaling and transport.^[5]^ The human caveolin-1 complex is composed of 11 protomers organized into a tightly packed disc with a flat membrane-embedded surface.^[6]^ It is involved in the regulation of multiple signaling pathways, such as the Ras, Akt, and MAPK pathways, and has been implicated in a variety of physiological processes, including lipid metabolism, cell proliferation, and apoptosis. Caveolin-1 has been shown to interacts with multiple proteins, including G protein-coupled receptors (GPCRs), ion channels, and signaling molecules, and modulates their function and localization. Recent studies have shown that Caveolin-1 can also interact with CD26 and modulate its signaling function. The interaction between Caveolin-1 and CD26 can induce changes in downstream signaling cascades triggered by small hormones, peptides, and light, making them important targets for drug discovery. Ohnuma et al demonstrated the interaction between CD26 and caveolin-1, suggesting its involvement in T cell costimulation and immune regulation.^[7]^ The binding of CD26 to caveolin-1 induces signaling events that lead to NF-κB activation, resulting in the upregulation of CD86 expression and subsequent T cell proliferation.^[8]^ The exact mechanisms by which caveolin-1 regulates signaling pathways in antigen-presenting cells and T cells require further investigation. In particular, targeting the Caveolin-1-CD26 interaction may have therapeutic potential.^[9]^ Therefore, elucidating the preferred conformations and key amino acids involved in CD26 and caveolin-1 interaction has significant scientific and clinical implications. Understanding the structure-function relationship and signaling pathways associated with this interaction will provide insights into protein-protein interactions and contribute to the development of targeted therapeutics. Moreover, the identification of potential therapeutic compounds that disrupt CD26 and caveolin-1 interactions may offer new strategies for treating diseases characterized by dysregulated immune responses.

A study conducted by Ohnuma et al provided evidence supporting the role of caveolin-1 as a ligand for CD26.^[10]^ Specifically, researchers discovered that CD26 expressed on activated memory T cells interacts with caveolin-1 present on monocytes loaded with tetanus toxoid. The interaction between CD26 and caveolin-1 in CD4^+^ T cells suggests their potential involvement in modulating immune responses.^[11]^ Activated memory T cells are key players in adaptive immune responses, and their interaction with monocytes, which are antigen-presenting cells, suggests a functional association between CD26 and caveolin-1 in the context of antigen recognition and presentation.^[12]^ One possible implication of this interaction is the regulation of T-cell activation and migration. CD26 has been implicated in T cell co-stimulation and the regulation of cytokine production, whereas caveolin-1 has been shown to play a role in T cell receptor signaling and immune synapse formation.^[13]^ The CD26-caveolin-1 interaction may provide an additional layer of regulation in these processes, contributing to the fine-tuning of immune responses. In addition to its role in immune responses, the CD26-caveolin-1 interaction is involved in various other functions: Cell adhesion and migration: Both CD26 and caveolin-1 have been implicated in cell adhesion and migration processes. The interaction between CD26 and caveolin-1 may play a role in cell adhesion and migration, contributing to cellular processes such as wound healing, tissue regeneration, and immune cell trafficking.^[14]^ Signal transduction: Caveolin-1 is involved in organizing and regulating signaling molecules in lipid rafts. By interacting with caveolin-1, CD26 may modulate the signal transduction pathways and cellular responses. This interaction can affect downstream signaling events triggered by various receptors, including T-cell receptors and other immune-related receptors.^[15]^ Lipid metabolism: Caveolin-1 is closely associated with lipid metabolism and cholesterol homeostasis. CD26, a membrane-bound protein, may be involved in lipid transport and metabolism via its interaction with caveolin-1. This interaction may affect lipid raft formation, lipid signaling, and lipid metabolism pathways in the cell.^[16]^ Membrane organization and vesicle trafficking: Caveolin-1 is a major component of caveolae, which are specialized membrane microdomains involved in membrane organization and vesicle trafficking.^[17]^ CD26 interaction with caveolin-1 may contribute to the formation and maintenance of caveolae structures as well as regulate vesicle trafficking processes within the cell. Moreover, CD26-caveolin-1 interaction has been implicated in several diseases. CD26 has been associated with immune-related disorders such as autoimmune diseases, inflammatory conditions, and cancer.^[18]^ Caveolin-1 dysregulation has been linked to various pathological conditions including cardiovascular diseases, neurodegenerative disorders, and cancer. The CD26-caveolin-1 interaction may play a role in the pathogenesis and progression of these diseases, making it a potential target for therapeutic interventions.

In this study, we used molecular dynamics simulations and alanine scanning to elucidate the key amino acid residues involved in the CD26-Caveolin-1 interaction and their contribution to binding affinity. Our results provide insights into the structural basis of the Caveolin-1-CD26 interaction and may facilitate the development of small-molecule inhibitors targeting this interaction. In silico studies are crucial for efficient drug discovery and the prediction of molecular interactions. For instance, studies have identified clemastatin B and other lignan derivatives as potential inhibitors of SARS-CoV-2. Erythrin, a lichen compound, showed strong anti-diabetic potential in molecular docking studies, suggesting that it is a safe and effective anti-diabetic agent.^[19,20]^ We used virtual screening of a Traditional Chinese Medicine Library (consisting of 1676 individual compounds) and an Anti-diabetic Compound Library (containing 147 compounds associated with diabetes development) databases based on the key amino acid residues of the Caveolin-1-CD26 interaction, and identified a potential lead compound (Crocin, Poliumoside, and Canagliflozin) for the development of immunomodulatory agents in the treatment of autoimmune diseases and cancer. Furthermore, drug-likeness and in silico absorption, distribution, metabolism, excretion, and toxicity (ADMET) studies, along with bioactivity prediction, will be employed in this study to evaluate the efficacy, safety, and pharmacokinetic profile of Crocin, Poliumoside, and Canagliflozin in early drug discovery.^[21–23]^

## 2. Materials and methods

### 2.1. Z-DOCK

ZDOCK 3.0.2 a software was used to simulate the docking between CD26 (PDB: 6b1e) and Caveolin-1(PDB: 7sc0). The structures of the proteins were prepared using the Protein Preparation Wizard in MOE 2015.10 (CCG, Ottawa, ON) and saved in PDB format. The input files for the docking process were generated using the ZDOCK Server, in which the search space and grid size were set to encompass the entire surface of the two proteins. The default scoring function of ZDOCK was used to calculate the binding affinity between the proteins.^[24]^ The top-ranked complexes were visually inspected and analyzed using PyMOL software to determine the key interacting residues and binding modes of the proteins.^[25]^

### 2.2. PDBePISA

To investigate the interaction between CD26 and Caveolin-1, we used PDBePISA to analyze the protein-protein interface and calculate the intermolecular interactions. The structures of CD26 and Caveolin-1 were obtained from the results of Z-DOCK and were uploaded to PDBePISA for analysis.^[26]^ The software identified the interface residues between the two proteins and calculated the intermolecular contacts, hydrogen bonds, and other types of interactions. The results of this analysis provided valuable information (ΔiG value and the interface area) on the nature and strength of the interactions between CD26 and Caveolin-1.

### 2.3. Molecular dynamics (MD) simulations and alanine scanning of CD26-Caveolin-1 complexes

Molecular dynamics simulations were performed using Gromacs 2022.3 (UU, SE) software.^[27]^ For the preprocessing of small molecules, AmberTools22 was employed to apply the GAFF force field, while Gaussian 16 W was used for hydrogenation and RESP potential calculation.^[28]^ The resulting potential data are incorporated into the topology file of the molecular dynamics system. The simulations were conducted under static conditions at a temperature of 300 K and an atmospheric pressure (1 Bar). An Amber99sb-ildn force field was utilized, and the solvent consisted of water molecules modeled using the Tip3p water model. To neutralize the total charge of the system, Na + ions were added as required. The energy minimization of the system was performed using the steepest descent method. This was followed by equilibration under an isothermal isovolumic ensemble (NVT) and isothermal isobaric ensemble (NPT) for 100,000 steps each. The coupling constant was set to 0.1 ps, and the equilibration duration was 100 ps. Subsequently, a free molecular dynamics simulation was conducted, consisting of 5,000,000 steps with a time step of 2 fs, resulting in a total simulation duration of 100 ns. Upon completion of the simulation, the built-in analysis tool of the software was used to analyze the trajectory. Key parameters, such as root-mean-square deviation (RMSD), root-mean-square fluctuation (RMSF), and protein rotation radius for each amino acid trajectory, were calculated. Additionally, the results were combined with free-energy calculations (MM/PBSA), free-energy topography, and other relevant data. To incorporate the alanine scanning technique, specific amino acid residues were systematically replaced with alanine, and subsequent molecular dynamics simulations were performed for each mutated system. Effect of alanine substitutions on RMSD, RMSF, and protein rotation radius. The number of hydrogen bonds, B-factor, and other relevant parameters were analyzed and compared with those of the wild-type system. This approach allowed the assessment of the functional importance of individual amino acids in protein structure and dynamics.

In molecular dynamics simulations using the MM/PBSA method, the parameters ΔGGAS, ΔGSOLV, and ΔTOTAL have the following meanings^[29]^:

ΔGGAS (change in gas-phase free energy): This represents the energy change owing to interactions when the molecule is in the gas phase. This energy term is typically obtained from molecular mechanics simulations, where denotes the energy change of the molecule in vacuum.

ΔGSOLV (change in solvation free energy): This represents the energy change owing to solvation effects. ΔGSOLV considers the energy change of a molecule in the presence of a solvent, accounting for processes such as molecular dissolution and interactions with solvent molecules. It is usually calculated using the Poisson-Boltzmann equation or other solvent models.

ΔTOTAL (total energy change): This denotes the overall energy change due to molecular interactions. ΔTOTAL is the sum of ΔGGAS and ΔGSOLV and represents the combined contribution of the gas phase and solvation effects.

The MM/PBSA calculation formula is as follows:

ΔTOTAL = ΔGGAS + ΔGSOLV

ΔGGAS was obtained from molecular mechanics simulations, while ΔGSOLV was calculated using a solvent model, typically employing the Poisson-Boltzmann equation. These energy change parameters were used to assess the contributions of molecular interactions and the stability of the molecules in different environments.

### 2.4. Prediction of binding sites

MOE 2015.10 (CCG) was used to identify the potential binding sites of the CD26-Caveolin-1 protein complex.^[30]^ The Receptor Grid Generation module of AutoDock Vina 2010 (Dr Oleg Trott, US) was employed to generate a docking grid file centered on the cocrystal ligand at the active site. Thirteen binding sites were predicted by MOE, and the top-ranked site, Hyd, was selected for further analysis. The Site Finder module identified 50, 1.96, 72, and 132 sites for PLB, Hyd, and Side, respectively, with the top-ranked sites selected for further receptor grid generation. The interaction site residues were determined to be CD26: GLU237 TYR241 TYR248 ARG147 GLY99 PHE98, as well as caveolin-1: F chain TYR151, F chain PHE160, G chain PHE160, H chain PHE160, I chain TYR148, and J chain TYR151 were also included in the site.

### 2.5. Preparation of protein targets and ligand libraries

The compounds used in this study were obtained from the Traditional Chinese Medicine Library (consisting of 1676 individual compounds) and the Anti-diabetic Compound Library (containing 147 compounds associated with diabetes development) (https://www.selleck.cn/screening/traditional-chinese-medicine-library.html and https://www.selleck.cn/screening/anti-diabetic-compound-library.html (accessed June 2, 2023)). The ligands and receptor grids were prepared using AutoDock Vina 1.5.6 in PyRx 0.8.^[31]^ A total of 1823 micromolecules in the format of 2D spatial data files (sdf) were processed using the LigPrep module, which generated the possible ionization states of the small molecules in the specified PH range (PH = 7.0 ± 2.0) under the condition of an OPLS4 force field. Eventually, the small molecules are transformed into 3D structures.

The CD26-Caveolin-1 complex structure from Z-DOCK was prepared using the Protein Preparation Wizard module in MOE 2015.10. This includes the addition of hydrogen atoms and residue side chains, structure optimization, and energy minimization. Water molecules were removed from the system, and the protonation states of the residues in the protein were generated under specific PH conditions (PH 7.0). Finally, the system was optimized under an OPLS4 force field to generate a more reasonable protein structure. Any impure molecules were removed from the model structures.

### 2.6. Receptor grid generation and virtual screening

AutoDock Vina 1.5.6, PyRx 0.8 was utilized for the virtual screening process. The pre-filtered compound library were screened against the CD26-Caveolin-1 complex using a grid box dimension of 81.26 × 73.44 × 66.14 Å³, centered at coordinates (73.27, 114.34, 69.32) Å to encompass the binding site of the complex. Compounds with binding energies greater than −9.00 kcal/mol were excluded from further consideration. A more stringent threshold was applied based on previous findings,^[32]^ indicating that −7.0 kcal/mol can effectively differentiate between specific and nonspecific protein-ligand interactions in AutoDock. The resulting docked ligands were visually inspected using PyMOL to select the most favorable binding positions.

To validate the results, known ligands and selected compounds were re-docked to the human CD26-Caveolin-1 complex using AutoDock Vina. Prior to molecular docking studies, the CD26-Caveolin-1 complex was modeled by employing the available CD26-Caveolin-1 structure as a template and using Modeller to address any missing residues. A grid box with the dimensions of 70.36 × 52.63 × 81.12 Å³ and center coordinates (85.64, 84.33, 57.65) Å were defined for the CD26-Caveolin-1 complex.

### 2.7. Characterisation of binding mechanism

The binding between CD26-Caveolin-1 and the compounds was evaluated and analyzed using AutoDock Vina 1.5.6.^[25]^

### 2.8. Molinspiration online tool and physicochemical properties of selected compounds

The bioactivity scores of Crocin, Poliumoside, and Canagliflozin were analyzed using the Molinspiration platform.^[33]^ This platform assigns numerical scores based on the likelihood that a compound exhibits specific biological activities. We evaluated the potential of these compounds as GPCR ligands, ion-channel modulators, kinase inhibitors, nuclear receptor ligands, protease inhibitors, and enzyme inhibitors. These scores provided insights into the bioactivity profiles of the compounds, aiding in the prediction of their potential therapeutic effects.

To forecast the physicochemical attributes of potential drug compounds, we used the SWISS ADME online resource (http://www.swissadme.ch/). The compounds are entered either via SMILES notation, file upload, or by sketching them. SWISS ADME will then analyze and predict multiple physicochemical properties, including XLOGP3, molecular weight, topological polar surface area, aqueous solubility, fraction of sp3 carbon atoms, rotatable bonds, hydrogen bond acceptors/donors, molar refractivity, guide drug design, and bioavailability assessment.

### 2.9. Druglikeness evaluation

Drug-likeness evaluation involves assessing a compound’s physicochemical properties and structural characteristics against predefined criteria to determine its potential as a safe and effective drug. In this study, the PreADMET tool (https://preadmet.qsarhub.com/druglikeness/) containing various rules, such as CMC_like, Lead_like, MDDR_like, Rule of Five, and WDI_like, was applied to evaluate a compound’s druglikeness. The CMC-like Rule, which is an extension of the Rule of Five (Ro5), assesses drug-likeness based on physicochemical properties, albeit with potentially modified or additional parameters compared with the original Ro5. On the other hand, the Lead-like Rule focuses on early stage drug discovery, identifying promising molecules for further development based on criteria such as molecular weight, lipophilicity, and hydrogen bonding capabilities. Meanwhile, the MDDR-like Rule is rooted in the Molecular Design Limited Database of Drug-like Compounds, evaluating drug-likeness based on its database criteria. Lastly, Lipinski’s Rule of Five (Ro5) remains the gold standard for predicting a compound’s ADME properties, emphasizing limits on hydrogen bond donors, acceptors, molecular weight, and octanol-water partition coefficient. Together, these rules aid in the selection of compounds with favorable pharmacokinetic properties for drug development.

### 2.10. ADMET prediction

The PreADMET tool can be utilized to forecast the absorption, distribution, metabolism, excretion, and toxicity (ADMET) characteristics of potential drug compounds. By submitting compound structures and choosing particular ADMET factors, this instrument employs computational algorithms and models to yield crucial information regarding a compound’s bioavailability, dissemination within the body, metabolic stability, excretion routes, and potential toxicological hazards. ADMET prediction involves a comprehensive evaluation of numerous aspects of a drug compound’s behavior, including gastrointestinal absorption, skin permeability, blood-brain barrier penetration, cell-based models for absorption and permeability, cytochrome P450 enzyme interactions, P-glycoprotein inhibition, mutagenicity, carcinogenicity, and acute fish toxicity. Taken together, these metrics offer significant insights into a drug’s effectiveness, safety, and environmental footprint, bolstering the drug development process and regulatory evaluations.

Various parameters are crucial for ADMET evaluation. AlogP98 indicates lipophilicity of a compound, which affects its bioavailability. Blood-Brain Barrier (BBB) and human colorectal adenocarcinoma cell line Caco-2 (Caco2) refer to the blood-brain barrier and a cell line, respectively, for studying intestinal absorption. CYP enzymes play a role in drug metabolism, whereas Human Intestinal Absorption, MDCK, and Skin_Permeability assess absorption and penetration abilities. Solubility, Plasma_Protein_Binding, and Solvation_Free_Energy affect drug distribution. Environmental and mutagenicity tests, such as algae_at, Ames_test, and hERG_inhibition, ensure safety. CarcinoMouse and CarcinoRat assessed the carcinogenicity risks. These factors collectively determine the efficacy and safety profile of a drug.

This study did not require an ethical review, so ethical approval was not necessary.

## 3. Results

### 3.1. The direct binding between CD26 and caveolin-1 exhibits two main conformational modes

**Figure 1.**
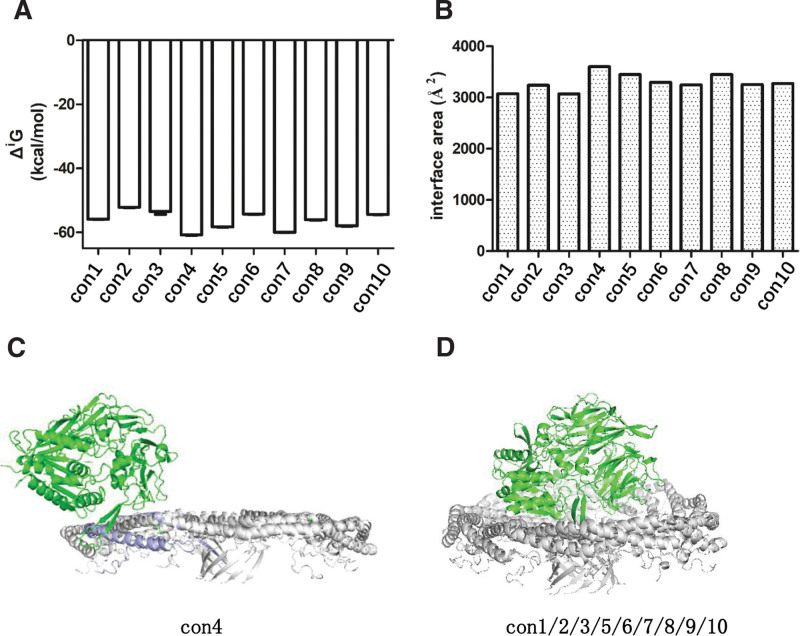
zDock docking results of CD26 and caveolin-1. (A) The ΔiG value indicates the gain in solvation free energy upon interface formation, expressed in kcal/mol. It is computed as the disparity between the total solvation energies of the isolated and interacting structures. A negative ΔiG signifies a hydrophobic interface or favorable protein affinity. This measurement does not encompass the contribution of established hydrogen bonds and salt bridges at the interface. (B) The interface area is quantified in Å2 and determined by subtracting the total accessible surface area of the isolated structures from that of the interacting structures, and then dividing the result by two. (C) CD26 binding to the J chain of caveolin-1’s scaffolding domain. (D) CD26 binding to the SR of caveolin-1, organized parallel to the membrane plane. SR = spoke region.

Z-DOCK simulation of CD26 and caveolin-1 docking generated 10 conformations with high scores. Among them, con4 exhibited significant differences from the other conformations. PDBePISA analysis revealed that con4 had the lowest ΔiG value, indicating a hydrophobic interface or favorable protein affinity (Fig. 1A). Additionally, con4 was ranked highest in terms of the interface area (Fig. 1B). In con4, CD26 was observed to bind to the J chain of caveolin-1’s scaffolding domain (Fig. 1C). This differs from the other conformations, represented by con1, where CD26 was found to bind to the spoke region (SR) of caveolin-1, which is organized parallel to the membrane plane (Fig. 1D). These findings suggest that con4 may represent a distinct binding mode between CD26 and caveolin-1, which is characterized by a hydrophobic interface and high protein affinity. The specific interaction between CD26 and the J chain of caveolin-1’s scaffolding domain in con4 highlights the potential importance of this region in its binding.

It is worth noting that the con1 of CD26-Caveolin-1 complex comprises 11 chains of caveolin-1 arranged in a tightly packed disc structure embedded in the membrane.^[34]^ The conformational differences observed between con1 and con4 may be attributed to the specific binding modes and interactions of CD26 with the different regions of caveolin-1. These findings highlight the dynamic nature of the CD26-Caveolin-1 interaction and provide insights into the flexibility of CD26 and its potential role in complex formation. Further studies focusing on the structural and functional implications of these flexible regions can contribute to a better understanding of the CD26-Caveolin-1 interaction and its significance in physiological and pathological processes. The identification of key amino acids involved in the CD26-Caveolin-1 interaction from the lowest free energy conformations provides valuable insights into the molecular basis of this interaction. These amino acids play crucial roles in establishing hydrogen bonds, π–π interactions, and cation–π interactions, thereby contributing to the stability and specificity of complex formation. Understanding these key interactions opens avenues for further research and drug design targeting the CD26-Caveolin-1 interaction, which has implications in disease mechanisms and therapeutic interventions.

### 3.2. Con1 exhibited higher thermodynamic stability compared to con4

**Figure 2.**
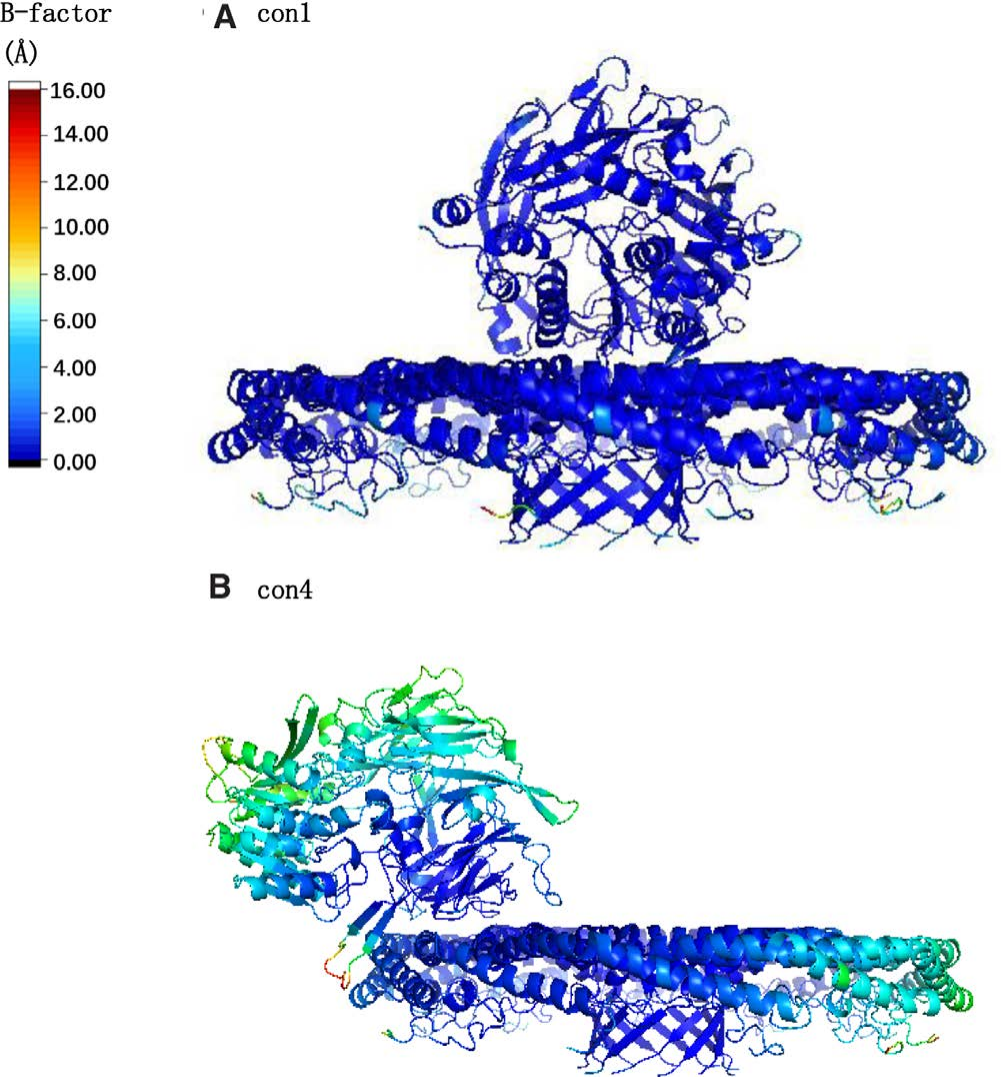
B-factor was utilized to demonstrate the thermal stability of protein region. The B-factor, calculated as the root mean square fluctuation (RMSF) from the molecular dynamics simulations, was used to illustrate the thermal stability of the CD26 and caveolin-1 protein complex. Blue coloring indicates higher thermodynamic stability, while red coloring indicates less stability. (A) con1: CD26 binding to the spoke region (SR) of caveolin-1; (B) con4: CD26 binding to the J chain of caveolin-1’s scaffolding domain. RMSF = root-mean-square fluctuation.

The thermal stability of CD26 and caveolin-1 protein complex was assessed using B-factor analysis, which calculates the root mean square fluctuation (RMSF) based on molecular dynamics simulations. The B-factor values were visualized to depict the thermal stability of different regions within the complex. In con1, where CD26 binds to the spoke region (SR) of caveolin-1, B-factor analysis revealed a higher level of thermodynamic stability, indicated by the predominance of blue coloring. This suggests that the con1 conformation maintains a more rigid and stable interaction between CD26 and the spoke region of caveolin-1 (Fig. 2A). Conversely, in con4, where CD26 binds to the J chain of caveolin-1’s scaffolding domain, the B-factor analysis displayed a greater level of fluctuation, as indicated by the presence of red coloring. This suggests that the con4 conformation exhibits a less thermodynamically stable interaction between CD26 and the J chain of caveolin-1’s scaffolding domain (Fig. 2B). Overall, these B-factor analyses provided valuable insights into the thermal stability and dynamic behavior of the CD26-Caveolin-1 interaction, highlighting distinct conformations and their implications for the stability and function of the protein complex.

Con1, where CD26 binds to the spoke region (SR) of Caveolin-1, exhibits higher thermodynamic stability. Thermal stability analysis revealed that con1 exhibited greater stability than con4, as evidenced by lower B-factor values and a predominance of blue coloring, indicating reduced fluctuations within the CD26-Caveolin-1 complex (Fig. 2).

### 3.3. Comparative analysis of con1 and con4 conformations in CD26-Caveolin-1 interaction dynamics

**Figure 3.**
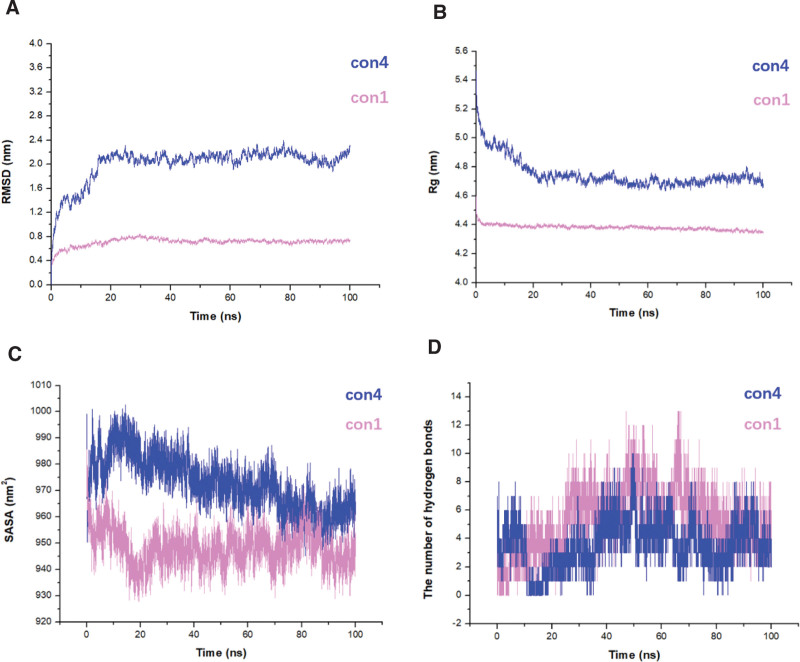
The two conformations of the CD26 and caveolin-1 complex reached equilibrium after 100 ns of molecular dynamics simulation. After completing the simulations, a comparative analysis of the trajectory of the two conformations (con1 and con4) of CD26 and caveolin-1 binding was performed. The trajectory of con4 is represented in blue, while the trajectory of con1 is represented in pink. (A) The RMSD of the amino acid trajectories; (B) The protein Rg; (C) The SASA; (D) The number of hydrogen bonds. Rg = gyration radius, RMSD = root-mean-square deviation, SASA = solvent-accessible surface area.

The root mean square deviation (RMSD) analysis (Fig. 3A) revealed that con4 exhibited an average RMSD value of 2 nm, whereas con1 had a significantly lower average RMSD value of only 0.5 nm. This suggested that the con1 conformation displayed more stable and less pronounced positional changes, indicating higher stability. The gyration radius (Rg) analysis, depicted in Figure 3B, demonstrated that the con1 protein structure had an average compactness of approximately 4.4 nm, which was notably superior to the average of 4.7 nm observed for con4. Furthermore, both conformations exhibited a consistent and stable decreasing trend in Rg throughout the simulations. Similarly, solvent-accessible surface area (SASA) analysis, presented in Fig. 3C) showed a stable decreasing trend for both con1 and con4 conformations. However, con1 exhibited smaller fluctuations in SASA than con4, further indicating its relatively higher stability. Moreover, hydrogen bond analysis revealed that con1 had a significantly higher number of hydrogen bonds than con4, particularly during the 15 to 80 ns simulation period (Fig. 3D). This suggests that the enhanced stability of con1 may be attributed to the contribution of molecular hydrogen-bonding interactions. In summary, analysis of RMSD, Rg, SASA, and hydrogen bond counts consistently demonstrated that con1 exhibited superior stability and conformational dynamics compared to con4 in the CD26-Caveolin-1 interaction. These findings highlight the potential significance of con1 for understanding the functional implications and therapeutic interventions associated with this interaction. MM/PBSA analysis also revealed energetic differences between the con1 and con4 conformations in the cd26-caveolin-1 interaction. The calculated ΔGGAS (gas phase energy contribution) and ΔGSOLV (solvent phase energy contribution) values provided insights into the binding free energy and stability of the two conformations. For con1, the ΔGGAS was determined to be 2888.95 kcal/mol, indicating a favorable energy contribution from the gas phase. In contrast, the ΔGSOLV was calculated as −2927.82 kcal/mol, indicating a significant favorable solvation energy contribution. Consequently, the overall ΔTOTAL for con1 was −38.85 kcal/mol, reflecting a thermodynamically stable and energetically favorable conformation. On the other hand, con4 exhibited a ΔGGAS of 2123.76 kcal/mol, suggesting a lower gas phase energy contribution compared to con1. Similarly, the ΔGSOLV for con4 was −2169.76 kcal/mol, indicating a less favorable solvation energy contribution. As a result, the overall ΔTOTAL for con4 was −46.01 kcal/mol, indicating a slightly lower stability and less favorable energetic profile compared to con1.

### 3.4. Comparison of amino acid fluctuations in con1 and con4 conformations of CD26-Caveolin-1 complex

**Figure 4.**
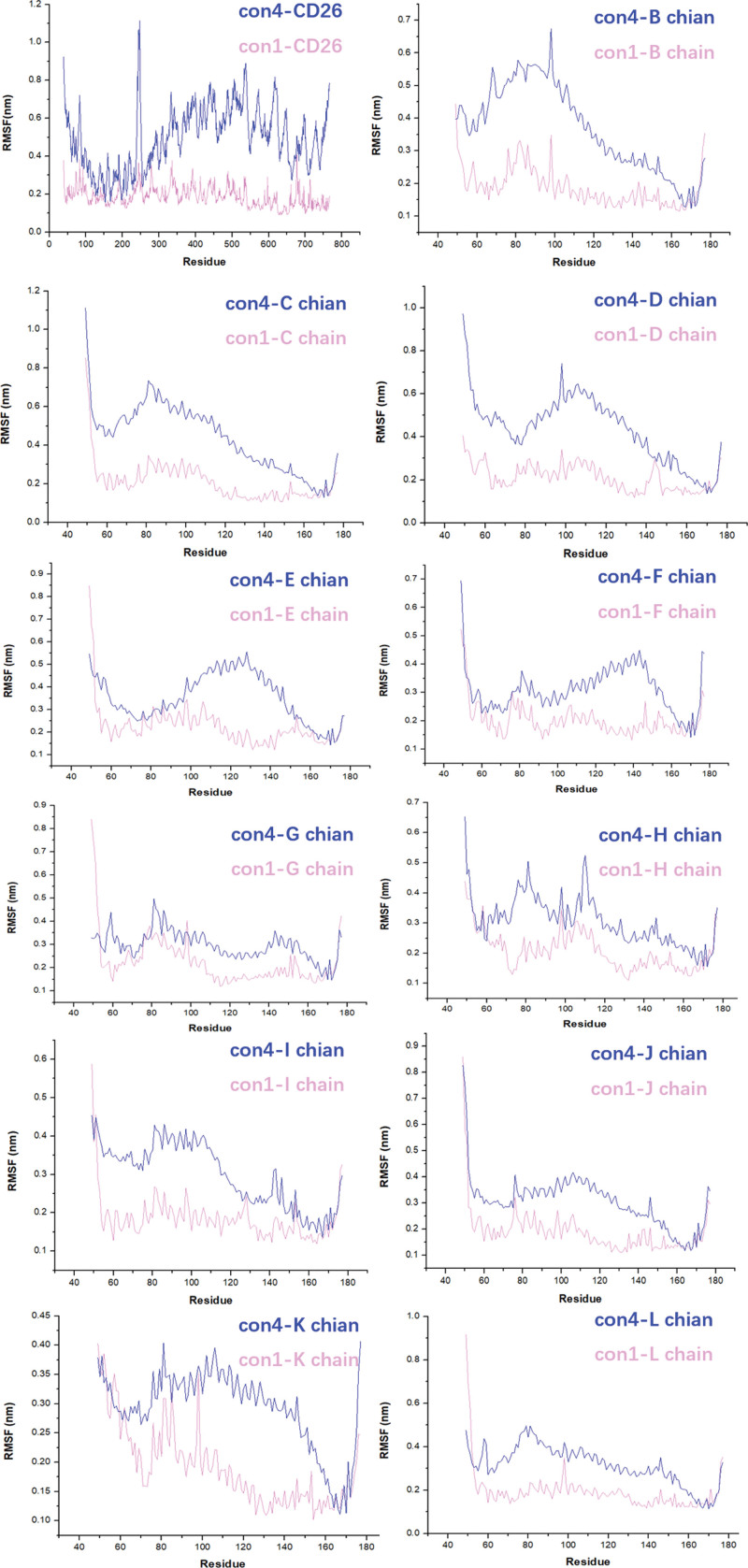
The RMSF values of the amino acid movement trajectories for con1 and con4. The human caveolin-1 complex (B-L chain) is composed of 11 protomers arranged in a tightly packed disc structure with a flat surface embedded in the membrane. RMSF = root-mean-square fluctuation.

Root-mean-square fluctuation (RMSF) analysis was performed to assess the movement of amino acid residues in the con1 and con4 conformations of the CD26-Caveolin-1 complex. The RMSF values represent the deviation of the amino acid positions from the reference structure, providing insights into the flexibility and dynamics of the complex. The results revealed that con1 exhibited generally lower RMSF values than con4, indicating reduced deviations of amino acid residues in con1 from their reference positions. Notably, the higher RMSF values in the CD26 regions of amino acids 200 to 250 and 650 to 700 indicate greater flexibility and conformational variability in these areas (Fig. 4). This flexibility can be attributed to the inherent dynamics and structural characteristics of CD26 and its interaction with caveolin-1. The higher RMSF values suggest that these regions are more susceptible to conformational changes and potential binding interactions.

### 3.5. Alanine scanning to identify key amino acids in CD26-Caveolin-1 interaction

**Figure 5.**
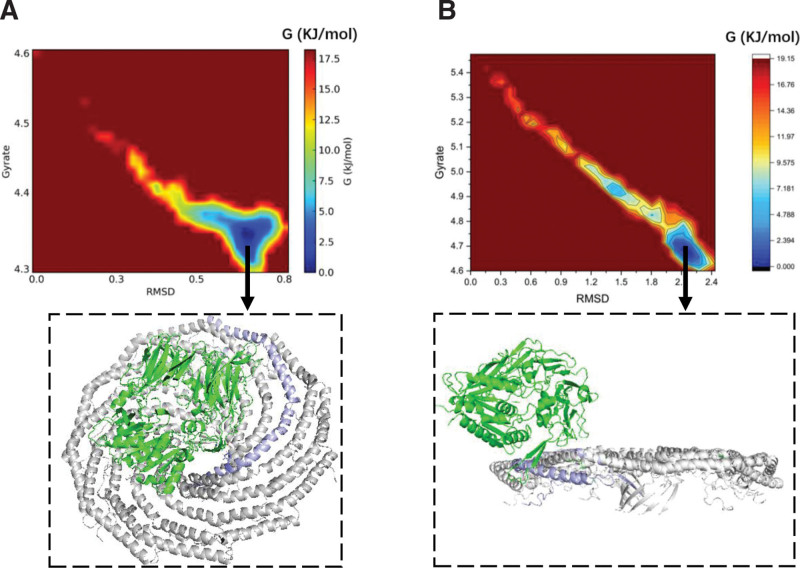
The Gibbs free energy landscape of con1 and con4. More stable conformations typically correspond to lower free energy regions, while less stable conformations correspond to higher free energy regions. Low free energy regions in the free energy landscape often represent energetically favorable conformations. (A) Extracting the Lowest Free Energy Conformation from the Gibbs Free Energy Landscape of con1; (B) Extracting the Lowest Free Energy Conformation from the Gibbs Free Energy Landscape of con4;

**Figure 6.**
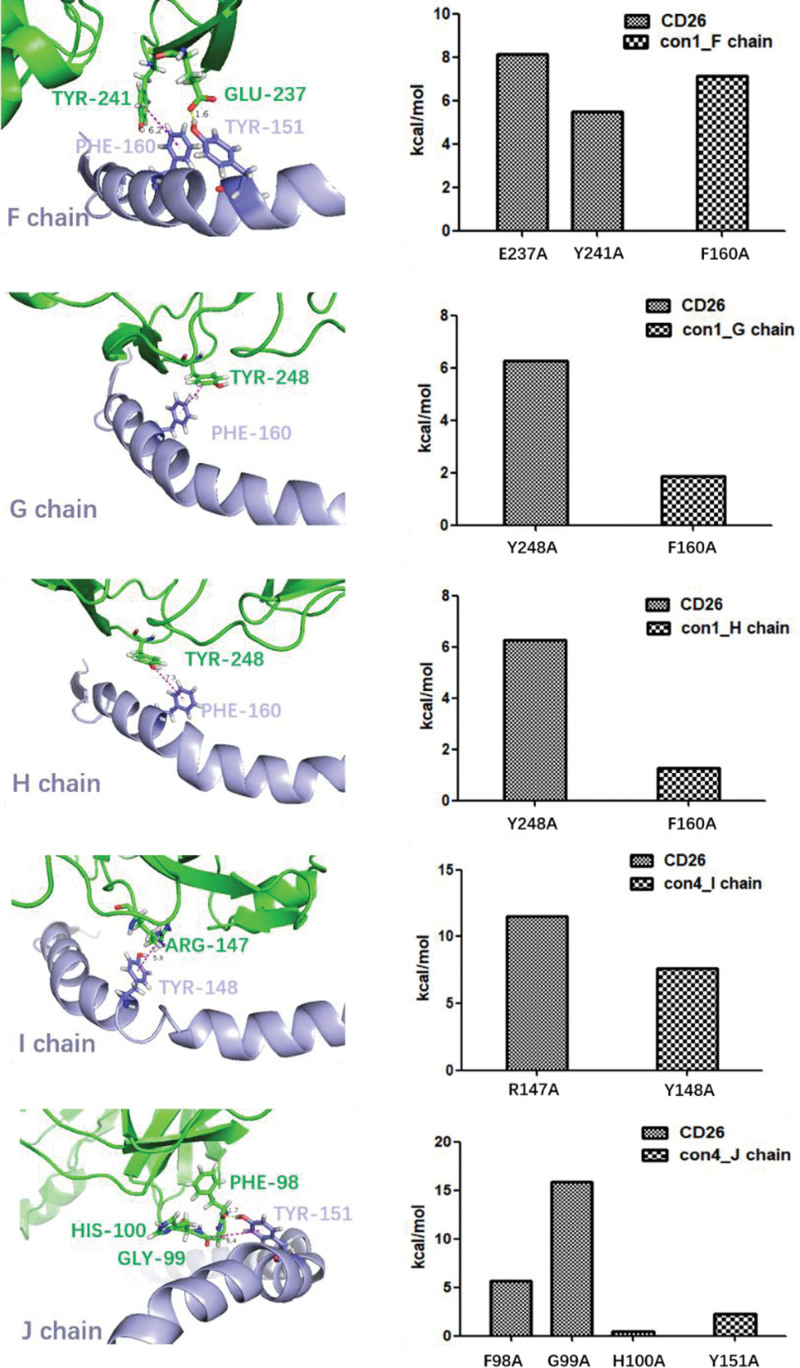
The conformation with the lowest free energy in con1 was extracted and analyzed using alanine scanning. In the simulation results of conformation 1, there are interactions observed between the F, G, H, I, and J chains of caveolin-1 and CD26. Specifically, CD26’s GLU237 forms hydrogen bond interaction with F chain’s TYR151, CD26’s TYR241 forms π–π interaction with F chain’s PHE160; CD26’s TYR248 forms π–π interaction with G chain’s PHE160; CD26’s TYR248 forms π–π interaction with H chain’s PHE160; CD26’s ARG147 forms π–π interaction with I chain’s TYR148; CD26’s GLY99 forms cation–π interaction with J chain’s TYR151; CD26’s PHE98 forms hydrogen bond interaction with J chain’s TYR151. Hydrogen bond interactions are represented by yellow dashed lines, while π–π interactions or cation–π interactions are depicted with purple dashed lines.

**Figure 7.**
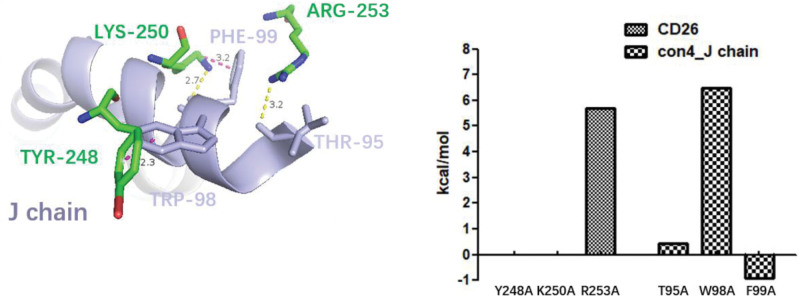
The conformation with the lowest energy in con4 was extracted and analyzed using alanine scanning. In the simulation results of conformation 4, there are interactions observed between the J chains of caveolin-1 and CD26. Specifically, CD26’s ARG253 forms hydrogen bond interaction with J chain’s THR95; CD26’s LYS250 forms a hydrogen bond with the J chain’s PHE99, while CD26’s LYS250 and the J chain’s PHE99 also engage in cation–π interactions; CD26’s TYR248 forms π–π interaction with J chain’s TRP98. Hydrogen bond interactions are represented by yellow dashed lines, while π–π interactions or cation–π interactions are depicted with purple dashed lines.

The Gibbs free energy landscapes of con1 and con4 were analyzed to extract the conformations with the lowest free energies, indicating their higher stability in the CD26-Caveolin-1 interaction (Fig. 5). The landscapes revealed that more stable conformations corresponded to lower free-energy regions, whereas less stable conformations corresponded to higher free-energy regions. The identification of low-free-energy regions in the landscapes signifies energetically favorable conformations for the CD26-Caveolin-1 complex. In con1, the lowest free energy conformation was extracted and further analyzed using 3alanine scanning. The simulation results revealed specific interactions between CD26 and the multiple chains of caveolin-1. CD26’s GLU237 formed a hydrogen bond (1.6 Å) with the F chain’s TYR151, CD26’s TYR241 engaged in a π–π interaction (6.2 Å) with the F chain’s PHE160, CD26’s TYR248 participated in π–π interactions (5.5 Å) with the G and H chains’ PHE160 (7.3 Å), CD26’s ARG147 formed a π–π interaction (5.9 Å) with the I chain’s TYR148, CD26’s GLY99 contributed to a cation–π interaction (6.4 Å) with the J chain’s TYR151, and CD26’s PHE98 formed a hydrogen bond (1.7 Å) with the J chain’s TYR151. These interactions indicate the involvement of key amino acids in stabilizing the CD26-Caveolin-1 complex within con1 (Fig. 6). Similarly, in con4, the lowest free-energy conformation was extracted and subjected to alanine scanning analysis. The simulation results demonstrated interactions primarily between the J-chain of caveolin-1 and CD26. Specifically, CD26’s ARG253 formed a hydrogen bond (3.2 Å) with the J chain’s THR95, CD26’s LYS250 interacted with the J chain’s PHE99 through hydrogen bonding (2.7 Å) and cation–π interactions (3.2 Å), and CD26’s TYR248 engaged in a π–π interaction (2.3 Å) with the J chain’s TRP98 (Fig. 7). These interactions highlight the importance of specific amino acids in stabilizing the CD26-Caveolin-1 complex within con4.

Alanine scanning analysis identified key amino acids that contribute to the stability of the CD26-Caveolin-1 complex in both con1 and con4 conformations.^[35]^ In con1, specific amino acids in CD26, such as GLU237, TYR241, TYR248, and ARG147, were found to interact with the F-J chain of Caveolin-1, establishing hydrogen bonds and cation or π–π interactions (Figs. 5A and 6). In con4, CD26 amino acids ARG253, LYS250, and TYR248 formed interactions with the J chain of Caveolin-1 through hydrogen bonds, cation–π interactions, and π–π interactions (Figs. 5B and 7). Furthermore, MM/PBSA analysis provides valuable insights into the energetic differences between con1 and con4 conformations in the CD26-Caveolin-1 interaction.^[36]^ Con1 exhibited specific interactions, including hydrogen bonds and π–π interactions, with key residues of caveolin-1, contributing to a stronger and more stable protein-protein complex. These favorable interactions likely contributed to the higher ΔGGAS and ΔGSOLV values and the overall thermodynamic stability observed for con1. In contrast, con4 showed a different binding mode to caveolin-1, involving interactions primarily with a different region of the protein. This alternative binding mode may result in a slightly weaker protein-ligand complex, leading to lower ΔGGAS and ΔGSOLV values and a slightly less favorable overall ΔTOTAL.

### 3.6. Virtual screening based on the key amino acids involved in the CD26-Caveolin-1 interaction

High-throughput virtual screening was conducted using a compound library that included both Traditional Chinese Medicine compounds (1676 compounds) and an Anti-diabetic Compound Library (147 compounds associated with diabetes development). The screening aimed to identify compounds that could potentially interact with key amino acids and modulate the CD26-Caveolin-1 interaction. Among the screened compounds, three candidates showed promising results. Crocin, Poliumoside, and Canagliflozin exhibited high docking scores and demonstrated favorable interactions with key amino acids at the CD26-Caveolin-1 interface. Crocin achieved a docking score of −14.18 kcal/mol, while polimoside achieved a docking score of −13.70 kcal/mol. Canagliflozin displayed a docking score of −9.780 kcal/mol (Table 1). Docking analysis revealed that these compounds formed interactions, such as hydrogen bonds and hydrophobic bonding, π–π interactions, and cation–π interactions, with the key amino acids involved in the CD26-Caveolin-1 interaction.

**Table 1:**
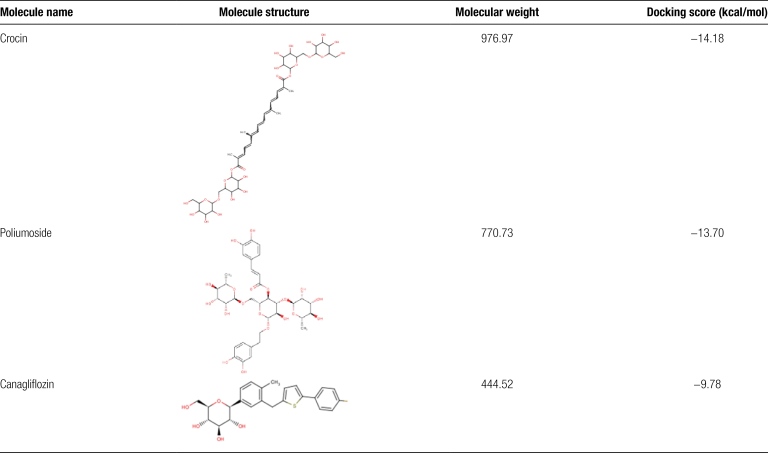
The chemical features and docking scores between CD26-Caveolin-1 and selected compounds.

**Figure 8.**
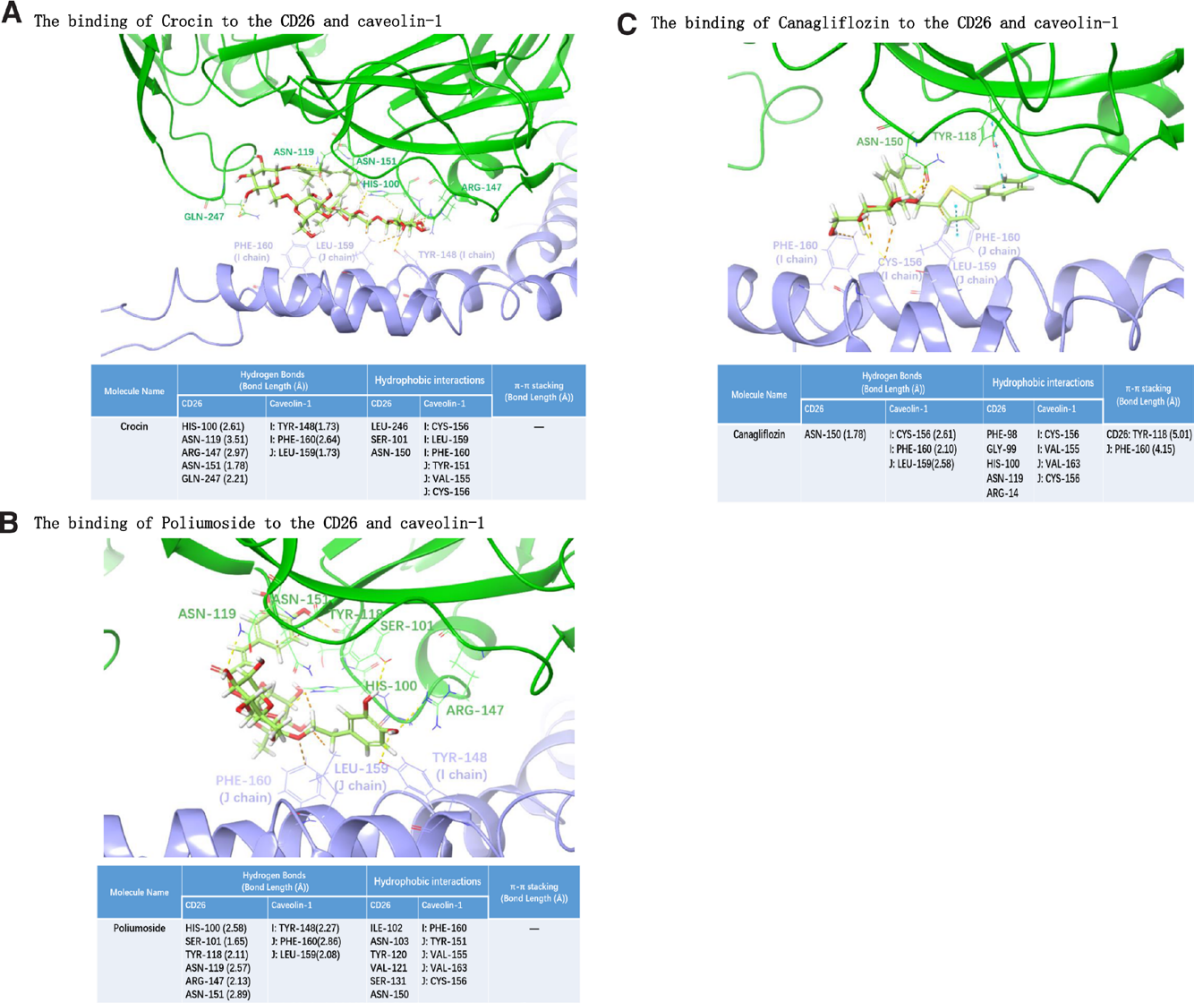
Using the conformation con1 of CD26 and caveolin-1 interaction as a template to perform virtual screening. A high-throughput virtual screening was performed on a Traditional Chinese Medicine Library (consisting of 1676 individual compounds) and an Anti-diabetic Compound Library (containing 147 compounds associated with diabetes development). The results revealed that Crocin, Poliumoside, and Canagliflozin exhibited high docking scores and demonstrated favorable interactions with the key amino acids involved in the CD26 and caveolin-1 interaction. (A) The binding of Crocin to the CD26 and caveolin-1 interface was analyzed to assess its interaction with the key amino acids involved, with docking score −14.18 kcal/mol; (B) The binding of Poliumoside to the CD26 and caveolin-1 interface was analyzed to assess its interaction with the key amino acids involved, with docking score −13.70 kcal/mol; (C) The binding of Canagliflozin to the CD26 and caveolin-1 interface was analyzed to assess its interaction with the key amino acids involved, with docking score −9.780 kcal/mol;

Specifically, crocin can form hydrogen bonds with several key amino acids in CD26, including HIS-100 (2.61 Å), ASN-119 (3.51 Å), ARG-147 (2.97 Å), ASN-151 (1.78 Å), and GLN-247 (2.21 Å). It also engages in hydrophobic interactions with LEU-246, SER-101, and ASN-150. On the other hand, Crocin can form hydrogen bonds with specific residues in Caveolin-1, specifically I chain TYR-148 (1.73 Å), I chain PHE-160 (2.64 Å), and J chain LEU-159 (1.73 Å). Additionally, it can interact hydrophobically with I chain CYS-156, I chain LEU-159, I chain PHE-160, J chain TYR-151, J chain VAL-155, and J chain CYS-156 (Fig. 8A).

Poliumoside can form hydrogen bonds with several key amino acids in CD26, including HIS-100 (2.58 Å), SER-101 (1.65 Å), TYR-118 (2.11 Å), ASN-119 (2.57 Å), ARG-147 (2.13 Å), ASN-151 (2.89 Å), and can establish hydrophobic interactions with ILE-102, ASN-103, TYR-120, VAL-121, SER-131, and ASN-150. Additionally, Poliumoside can form hydrogen bonds with Caveolin-1’s I chain TYR-148 (2.27 Å), J chain PHE-160 (2.86 Å), and J chain LEU-159 (2.08 Å), and can engage in hydrophobic interactions with Caveolin-1’s I chain PHE-160, J chain TYR-151, J chain VAL-155, J chain VAL-163, and J chain CYS-156 (Fig. 8B).

Canagliflozin can form hydrogen bonds with several key amino acids in CD26, including ASN-150 (1.78 Å), and establish hydrophobic interactions with PHE-98, GLY-99, HIS-100, ASN-119, and ARG-147. It also forms a π–π interaction with TYR-118 (5.01 Å). On the other hand, canagliflozin can form hydrogen bonds with Caveolin-1’s I chain CYS-156 (2.61 Å), I chain PHE-160 (2.10 Å), and J chain LEU-159 (2.58 Å), and engage in a π–π interaction with the J chain PHE-160 (4.15 Å). Additionally, it formed hydrophobic interactions with Caveolin-1’s I chain CYS-156, I chain VAL-155, J chain VAL-163, and J chain CYS-156 (Fig. 8C).

### 3.7. Bioactivity prediction for candidate small molecules

Based on the bioactivity prediction results obtained through Molinspiration, Crocin, Poliumoside, and Canagliflozin exhibited varying potential as ligands or inhibitors for different biological targets (Table 2). Crocin showed the least potential as a GPCR ligand, ion channel modulator, kinase inhibitor, nuclear receptor ligand, protease inhibitor, and enzyme inhibitor, with negative scores across all categories. Poliumoside demonstrated slightly higher scores, indicating modest potential, especially as a kinase and nuclear receptor ligand. In contrast, canagliflozin displayed the highest scores, particularly as a GPCR ligand, kinase inhibitor, and enzyme inhibitor, suggesting stronger bioactivity potential.

**Table 2:** Molinspiration bioactivity scores for crocin, poliumoside, and canagliflozin.

Molinspiration bioactivity score	Crocin	Poliumoside	Canagliflozin
GPCR ligand	−3.52	−1.13	0.15
Ion channel modulator	−3.68	−2.22	−0.21
Kinase inhibitor	−3.67	−1.80	0.15
Nuclear receptor ligand	−3.65	−1.82	0.07
Protease inhibitor	−3.24	−0.75	0.02
Enzyme inhibitor	−3.53	−1.39	0.33

GPCR ligand = G-Protein Coupled Receptor Ligand.

### 3.8. Physicochemical properties and druglikeness evaluation

Based on the physicochemical properties and drug-likeness evaluation results (Tables 3 and 4), we can draw some conclusions about the selected compounds. Despite their large molecular weights and high TPSA values, crocin and poliumoside do not fully comply with the standard druglikeness criteria, such as the Rule of Five or the Lead-like Rule. This suggests that they may face challenges in terms of their bioavailability and pharmacokinetic properties. In contrast, canagliflozin meets the criteria for the CMC_like_Rule and is considered suitable under the Rule of Five, indicating a higher potential for druglikeness. However, it violates the Lead_like_Rule, possibly because of its smaller size and fewer rotatable bonds than the other two compounds.

**Table 3:** Physicochemical properties of selected compounds.

Compound	XLOGP3	MW (g/mol)	TPSA (Å^2^)	Log S (ESOL)	Fraction Csp3	Rotatable bonds	Hydrogen bond acceptors	Hydrogen bond donors	Molar refractivity
Crocin	−2.49	976.96	391.20	−3.01	0.64	20	24	14	227.19
Poliumoside	−1.60	770.73	304.21	−2.92	0.57	13	19	11	179.64
Canagliflozin	3.23	444.52	118.39	−4.71	0.33	5	6	4	116.75

Fraction Csp3 = Fraction of sp3-hybridized Carbon atoms, Log S (ESOL) = Logarithm of Solubility (Estimated SOLubility), MW (g/mol) = Molecular Weight, TPSA (Å2) = Topological Polar Surface Area(in Ångströms squared), XLOGP3 = Extended LogP Calculator Version 3.

**Table 4:** Druglikeness evaluation of screened compounds.

Compound	CMC_like_Rule	Lead_like_Rule	MDDR_like_Rule	Rule_of_Five
Crocin	Not qualified	Violated	Drug-like	Violated
Poliumoside	Not qualified	Violated	Drug-like	Violated
Canagliflozin	Qualified	Violated	Mid-structure	Suitable

CMC_like_Rule: A set of criteria similar to drug-likeness, assessing whether molecules ossess characteristics that make them potential drug candidates.

Lead_like_Rule: a set of criteria used to evaluate whether lead compounds, which show some biological activity, have the potential for further development and optimization in the drug discovery process.

MDDR_like_Rule: Molecular Design Data Repository. Criteria based on molecular characteristics from this database, used to assess whether new molecules share similar properties with known drug molecules or biologically active molecules.

Rule_of_Five: The Rule of Five includes five main criteria: a molecular weight less than 500 Daltons, no more than 5 hydrogen bond donors, no more than 10 hydrogen bond acceptors, a logP value (a measure of lipophilicity) not greater than 5, and no more than 10 rotatable bonds. Molecules that meet these criteria are more likely to have favorable pharmacokinetic properties and oral bioavailability.

### 3.9. ADMET prediction

The summarized ADMET predictions offer valuable insights into the pharmacokinetic and toxicological profiles of crocin, polimoside, and canagliflozin. Canagliflozin, with its higher BBB and Caco2 permeability values, has better absorption and potential to cross biological membranes, which is crucial for effective drug delivery (Table 5). However, it also raises concerns owing to its mutagenicity in the Ames test and medium risk for human Ether-à-go-go Related Gene (hERG) inhibition, suggesting a need for closer toxicological evaluation. In contrast, crocin and polimoside exhibit lower solubility and permeability, which might hinder their bioavailability and distribution within the body. Furthermore, the positive carcinogenicity results observed in mice for both crocin and polimoside underscore the importance of careful safety assessment. The CYP inhibition profiles of these compounds also deserve attention, as they can affect drug metabolism and potentially lead to drug–drug interactions.

**Table 5:** Summary of absorption, distribution, metabolism, excretion and toxicity predictions of hit compounds.

ADMET parameters	Crocin	Poliumoside	Canagliflozin
AlogP98_value	−2.722	−0.476	3.4466
AMolRef	233.4858	178.199	117.9672
BBB	0.0272759	0.0283904	0.240969
Buffer_solubility_mg/L	9.51883	834.98	19.0607
Caco2	11.0509	8.91077	6.4235
CYP_2C19_inhibition	Inhibitor	Inhibitor	Inhibitor
CYP_2C9_inhibition	Inhibitor	Inhibitor	Inhibitor
CYP_2D6_inhibition	Non	Non	Non
CYP_2D6_substrate	Non	Non	Non
CYP_3A4_inhibition	Inhibitor	Inhibitor	Inhibitor
CYP_3A4_substrate	Weakly	Weakly	Substrate
HIA	0.021798	1.823753	90.35583
MDCK	0.0628463	0.0434188	2.27653
Pgp_inhibition	Non	Non	Non
Plasma_Protein_Binding	22.36946	45.47512	90.41183
Pure_water_solubility_mg_L	568.439	113.675	6.63389
Skin_Permeability	−2.33425	−3.17333	−3.8945
Solvation_Free_Energy	−72.4	−65.08	−21.590000
algae_at	0.00192344	0.00041769	0.00626256
Ames_test	Non-mutagen	Non-mutagen	Mutagen
Carcino_Mouse	Positive	Negative	Negative
Carcino_Rat	Positive	Negative	Negative
daphnia_at	5.53028	0.602695	0.0363928
hERG_inhibition	Ambiguous	Ambiguous	Medium_risk
medaka_at	78.6123	0.889446	0.00303488
minnow_at	96.7506	1.4567	0.00917162
TA100_10RLI	Negative	Negative	Negative
TA100_NA	Negative	Negative	Positive
TA1535_10RLI	Negative	Negative	Positive
TA1535_NA	Negative	Negative	Negative

Ames_test = A test used to assess the mutagenicity of a compound by checking its ability to induce mutations in bacteria, BBB = Blood-Brain Barrier, Caco2 = human colorectal adenocarcinoma cell line Caco-2, CYP2C19, CYP2C9, CYP2D6, CYP3A4 = refer to the specific cytochrome P450 enzymes which are involved in drug metabolism, hERG_inhibition = Refers to the inhibition of the human Ether-à-go-go Related Gene (hERG) potassium channel, which can lead to cardiac arrhythmias. Important in drug safety evaluation, HIA = Human Intestinal Absorption, MDCK = Madin-Darby Canine Kidney cell line, minnow_at = Toxicity tests using different aquatic organisms (algae, daphnia, medaka fish, and minnows) to assess the environmental impact of a compound, Pgp_inhibition = Refers to the inhibition of P-glycoprotein. algae_at, daphnia_at, medaka_at, TA100_10RLI, TA100_NA, TA1535_10RLI, TA1535_NA = Specific strains or conditions used in mutagenicity testing.

## 4. Discussion and conclusion

Previous studies have suggested that the interaction between CD26 and caveolin-1 is mediated by specific amino acid regions, namely CD26 residues 201 to 211 and caveolin-1 residues 82 to 101. However, the presence of 11 protomers in caveolin-1 introduces complexity and challenges in accurately characterizing this interaction. The involvement of multiple protomers may lead to variations in the binding interface and potentially hinder screening of therapeutic interventions. Therefore, further research is warranted to precisely define the amino acids involved in the CD26-caveolin-1 interaction, taking into account the structural arrangements of the caveolin-1 protomers. This will enhance our understanding of the interactions and facilitate more accurate screening of drugs targeting this pathway. Both studies demonstrated the potential of virtual screening in drug discovery, identifying flavonoids and urushiol derivatives as promising HDAC2 inhibitors through molecular docking, MD simulations, and binding free energy calculations, highlighting the importance of computational methods in finding novel drug candidates.^[37,38]^ Our findings shed light on the conformational modes, thermodynamic stability, dynamics, and key amino acids involved in the CD26-Caveolin-1 interaction. The CD26-Caveolin-1 complex exhibited two main conformational modes, namely con4 and con1/2/3/5/6/7/8/9/10 (Fig. 1C and D). Because of the similarity of conformations among con1/2/3/5/6/7/8/9/10, con1 was selected as the representative protein for further investigation. Con4, characterized by CD26 binding to the J-chain of Caveolin-1’s scaffolding domain, represents a distinct binding mode with a hydrophobic interface and high protein affinity. Comparative analysis of con1 and con4 revealed that con1 displayed a higher thermodynamic stability, conformational dynamics, compactness, and a greater number of hydrogen bonds (Figs. 3 and 4). These characteristics suggest that con1 may play a crucial role in the functional implications and therapeutic interventions associated with the CD26-Caveolin-1 interaction.

Virtual screening based on the key amino acids involved in the CD26-Caveolin-1 interaction identified three potential compounds, Crocin, Poliumoside, and Canagliflozin, which showed promising results in terms of high docking scores and favorable interactions with the key amino acids at the CD26-Caveolin-1 interface (Fig. 8). These compounds engaged in hydrogen bonds, hydrophobic interactions, and π-π interactions with critical residues in both CD26 and Caveolin-1, suggesting their potential as modulators of the CD26-Caveolin-1 interaction. Further experimental validation and studies are necessary to explore the efficacy and mechanisms of action of these compounds in modulating the CD26-Caveolin-1 interaction and their potential applications in disease treatment.

It is indeed surprising that there have been limited studies specifically investigating the key amino acids involved in the CD26-caveolin-1 interaction. Given the importance of this interaction in various biological processes, further research will provide valuable insights into the molecular mechanisms and functional implications of this interaction. In summary, this study provides valuable insights into the conformational modes, thermodynamic stability, dynamics, and key amino acids involved in the CD26-Caveolin-1 interaction. The distinct conformations, con1 and con4, exhibit differential stability, dynamics, and interactions, highlighting the potential functional implications of these modes. The identified key amino acids and their interactions can serve as targets for further studies and development of therapeutic interventions.

The virtual screening results presented potential compounds that can modulate the CD26-Caveolin-1 interaction, paving the way for future drug discovery efforts targeting this pathway. Furthermore, bioactivity prediction, physicochemical properties, drug-likeness evaluation, and ADMET predictions were comprehensively analyzed for crocin, polimoside, and canagliflozin. Canagliflozin showed the highest bioactivity potential, particularly as a GPCR ligand and kinase inhibitor, and exhibited better absorption with higher BBB and Caco2 permeability. However, its mutagenicity and the medium risk of hERG inhibition require closer toxicological assessment. Conversely, crocin and polimoside, despite showing modest bioactivity, face challenges due to their physicochemical properties and noncompliance with druglikeness criteria, indicating potential bioavailability and pharmacokinetic issues. Furthermore, carcinogenicity concerns have arisen for crocin and polimoside. These findings provide a holistic view of the efficacy, safety, and pharmacokinetic profiles of the compounds, aiding early drug discovery decisions (Tables 2–5).

Although computational studies provide valuable insights in computer-aided drug design, they have limitations that necessitate rigorous experimental validation. Despite their sophistication, computational models often rely on assumptions and approximations that may not fully reflect the intricacies of biological systems. The accuracy of these models depends heavily on the quality and diversity of the datasets used for training, which may introduce biases or inaccuracies if they are not sufficiently comprehensive. Furthermore, the dynamic and interactive nature of biological processes may not be fully captured using static computational models. Therefore, further validation through in vitro and in vivo studies is warranted before clinical translation can be realized.^[39,40]^

In conclusion, this study offers deep insights into the CD26-Caveolin-1 interaction, identifying key amino acids and distinct conformational modes. Virtual screening revealed promising compounds, with canagliflozin showing high bioactivity and favorable physicochemical properties. However, its mutagenicity and risk of hERG inhibition necessitate careful toxicological assessment. Despite their bioactivity, crocin and poliumoside have bioavailability and pharmacokinetic concerns along with carcinogenicity risks. These results provide a comprehensive evaluation of early drug discovery.

## Author contributions

**Conceptualization:** Xingkui Xue.

**Data curation:** Xiaopeng Hu, Chunmei Jiang, Yanli Gu.

**Formal analysis:** Xiaopeng Hu, Yanli Gu.

**Funding acquisition:** Xingkui Xue.

**Methodology:** Xiaopeng Hu.

**Project administration:** Xiaopeng Hu.

**Software:** Chunmei Jiang.

**Writing – original draft:** Xiaopeng Hu.

**Writing – review & editing:** Xingkui Xue.
